# Cilia-driven flows in the brain third ventricle

**DOI:** 10.1098/rstb.2019.0154

**Published:** 2019-12-30

**Authors:** Gregor Eichele, Eberhard Bodenschatz, Zuzana Ditte, Ann-Kathrin Günther, Shoba Kapoor, Yong Wang, Christian Westendorf

**Affiliations:** 1 Max Planck Institute for Biophysical Chemistry, Am Fassberg 11, 37077 Göttingen, Germany; 2 Max Planck Institute for Dynamics and Self-Organization, Am Fassberg 17, 37077 Göttingen, Germany

**Keywords:** cerebrospinal fluid, extracellular vesicles, fluid dynamics, translational polarity, hypothalamus, tanycyte

## Abstract

The brain ventricles are interconnected, elaborate cavities that traverse the brain. They are filled with cerebrospinal fluid (CSF) that is, to a large part, produced by the choroid plexus, a secretory epithelium that reaches into the ventricles. CSF is rich in cytokines, growth factors and extracellular vesicles that glide along the walls of ventricles, powered by bundles of motile cilia that coat the ventricular wall. We review the cellular and biochemical properties of the ventral part of the third ventricle that is surrounded by the hypothalamus. In particular, we consider the recently discovered intricate network of cilia-driven flows that characterize this ventricle and discuss the potential physiological significance of this flow for the directional transport of CSF signals to cellular targets located either within the third ventricle or in the adjacent hypothalamic brain parenchyma. Cilia-driven streams of signalling molecules offer an exciting perspective on how fluid-borne signals are dynamically transmitted in the brain.

This article is part of the Theo Murphy meeting issue ‘Unity and diversity of cilia in locomotion and transport’.

## 1. Introduction

Greek physicians already knew about the liquor-filled delicate cavities (the ‘ventricles’) inside the human brain and considered them as the seat of mental capacity, including cognition, memory, awareness and imagination. The physician philosophers thought that the ‘Common’ and the ‘Imaginative’ senses reside in the two large lateral ventricles located mirror-symmetrically in the front of the brain. The bipartite middle ventricle would encompass the ‘Estimative’ and ‘Phantastic’ senses. The last ventricle in the back of the brain would be devoted to the ‘Memorative’ sense [1]. Modern neurobiology places mental functions right into the dense tissue of the brain composed of neurons, nerve fibres and glia cells.

**Figure 1.**
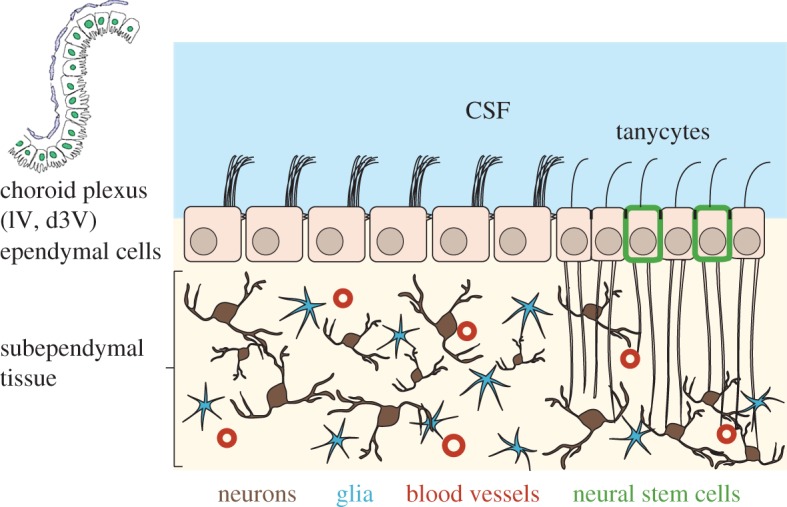
Scheme of the anatomy of the ventral part of the third ventricle (v3V). The lateral ventricles (lV), the dorsal part of the third ventricle (d3V) and the fourth ventricle (4V) contain a choroid plexus (CP) that secretes CSF. Propelled by beating cilia bundles located at the apical side of ependymal cells, CSF partitions above the ependymal cell layer in a complex manner (figure 3*a*). Subependymal neurons may be scattered or form clusters (nuclei) that carry out specific functions such as control of circadian timing or control of energy metabolism. In the v3v, the ependymal layer contains tanycytes, which are specialized glia cells that are bi-ciliated and send long processes that contact neurons, glia and blood vessels in the subependymal brain tissue. Some of the tanycytes have stem cell properties. CSF and interstitial fluid can pass between ependymal cells, while tanycytes have occluding junctions that form a seal preventing passage of solutes and water.

Despite their ‘downgrading’, brain ventricles continue to attract attention, perhaps more now than in the past when physiologists viewed them primarily as toxic waste dumps for metabolites that the brain eliminated. Contemporary neurobiologists view the cerebrospinal fluid (CSF)-filled ventricles as dynamic reservoirs of signalling substances and nutrients that can reach neurons, glia cells and stem cells (figure 1). This way of looking at ventricles leads to a host of questions. Which signalling molecules are present in the CSF? How do they interact with the various ependymal and subependymal cell types? How are signals taken up by cells? Which physiological processes does uptake trigger? Are CSF components delivered uniformly to all nearby brain tissues or is substance delivery targeted to a specific location? With regard to the last question, Faubel *et al.* [2] have identified a cilia-propelled network of CSF streams that run along the walls of the ventricle. These streams may transport factors in CSF to particular ependymal or subependymal tissue regions. It was known that motile cilia propel CSF along the ventricular surface [3,4] but not that flows form a system of interwoven streams.

The aim of this brief review is to provide background knowledge for selected components of the ventricular system (figure 1), and information that should be helpful for designing experiments that address some of the above raised questions.

## 2. Brain ventricles

The central nervous system of vertebrate animals develops from the neural tube. The wall of the tube consists of neuronal progenitor cells and encloses the CSF-filled ventricle. Through a combination of proliferation, migration and differentiation of the progenitor cells, fore-, mid- and hindbrain form at the front end of the neural tube, while the back end of the neural tube gives rise to the spinal cord. Brain ventricles co-develop with the nervous system so that around the time of birth, four distinct brain ventricles have formed. In the postnatal brain, ventricles are enclosed by the ependyma that consists mostly of E1 cells (ependymal cells, ependymocytes, figure 1; [5,6]). E1 cells derive from embryonic radial glia cells [7,8] and carry a characteristic apical bundle of motile cilia that coordinately beat and in this way propel CSF along the ventricular wall (figure 1).

**Figure 2.**
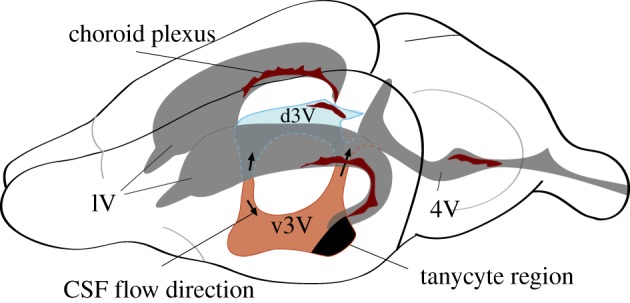
The four CSF-filled ventricles in the adult mouse brain. Highlighted in colour are the dorsal (d3V, blue) and ventral (v3v, light brown) parts of the third ventricle. The two lateral ventricles feed via a canal into the mid-plane located 3V. At the site of junction, CSF flows either dorsally (up arrow) into the d3V or ventrally (down arrow) into the v3v. At their back end, d3V and v3v connect via the aqueduct into 4V. Most of the lining of the ventricles consists of ependymal cells (E1 cells). The dark-shaded area in v3v consists primarily of α- and β-tanycytes. Dark brown features represent the secretory epithelium of the choroid plexi that releases CSF and secretes a great variety of small and macromolecular solutes. Dorsal is on the top and anterior to the left.

The shape of the ventricular cavity in brains from different species varies enormously to match the diversity of brain anatomy [9]. Most vertebrates have four ventricles except for teleost fishes, which have three. Ventricles are serially docked and interconnect through interventricular foramina. In rodents (figure 2), the two crescent-shaped lateral ventricles (lV) are each embedded in one of the forebrain hemispheres and connect, via a pair of interventricular passages, to the third ventricle (3V). The narrow 3V is squeezed in between the left and right halves of the diencephalon. In rodents, 3V is separated into a ventral (v3v) and a dorsal (d3V) part. The ventral part resides between the right and the left halves of the hypothalamus. Posteriorly, 3V connects through the aqueduct to the fourth ventricle (4V) that is surrounded by the mid- and hindbrain tissue. 4V connects to both the central canal of the spinal cord and to the subarachnoid space. The latter surrounds the brain and spinal cord. CSF exits the subarachnoid space through arachnoid granulations and in this way flows into the vascular system [10]. An additional exit path for CSF is the perineural flux across the cribriform plate and from there on to the nasal mucosa [11].

## 3. Choroid plexus, a source of signalling factors and extracellular vesicles

The intensely vascularized structure of the *plexus choroïdes* or choroid plexus (CP) inside the lateral ventricles of the human brain is shown in the detailed anatomical drawings by Vesalius [12]. This particular CP had already been identified in antiquity as a worm-like structure. There is a CP in each of the four ventricles that continuously produces, in humans, about 500 ml of CSF per day [13]. The continuous production of CSF contributes to a steady ventricular CSF flow. Heartbeat, breathing and body motion also contribute to CSF flow [10,14–17]. In rodents, the v3v has narrow entry and exit ducts and the wall-to-wall distance measures only 100–200 µm. Therefore, the major driving force for the flow is likely to be the beating of cilia bundles.

In the brain of lampreys, a primitive vertebrate animal, the CP distributes throughout the ventricles [18]. In mammals [19], CP is present in each of the four ventricles at specific locations (figure 2). Histologically, the CP consists of a secretory epithelium that encloses a web of fenestrated blood vessels. CP produces and releases CSF and electrolytes into the ventricles [20]. In addition, CP also provides various micronutrients, hormones, neurotransmitters, neurotrophins, peptide hormones, such as melanin-concentrating hormone, and growth factors that occur in CSF and in some cases are secreted in an age-dependent manner [21–25]. These agents are either directly synthesized by CP epithelial cells or enter the CP as components of the blood that pass through the fenestrated capillaries. Tight junctions connect the CP epithelial cells that form the blood–CSF barrier. Thus, a passage of substances into the ventricles requires selective trans-epithelial transporters. Such transporters are abundantly expressed in CP epithelial cells [26]. CSF constituents also enter into the ventricular space from the interstitial fluid-containing parenchyma. Lastly, there is release of neuropeptides and neurotransmitters from CSF-contacting neurons or neurons whose axonal terminals contact the CSF [27]. Some of the CSF hormones modulate cilia beating frequency and thus influence CSF flow [22].

The CSF also contains extracellular vesicles (EVs) [28,29]. EVs are membrane vesicles of a diameter between 30 and 150 nm and are of endocytic origin. Most cell types secrete EVs. EVs contain cellular proteins, small molecules and nucleic acids such as miRNA and non-coding RNAs [30–32]. EVs are likely to play a role in intercellular communication, in pathogenesis and are a reservoir of biomarkers and may also be in a pathway by which cells pass on unwanted materials. EVs play a role in different stem cell niches such as the mesenchymal stem cell niche, cancer stem cell niche and the pre-metastatic niche [33–35]. A community compendium for EVs is found in databases such as Vesiclepedia [36,37] and ExoCarta [38–40].

Adult neurogenic stem cell niches in which neurogenesis persists after birth are found in the subventricular zone of the lateral ventricle, in the subgranular zone of the dentate gyrus and along and beneath the v3v wall [8,24,41,42]. It has been hypothesized [43] that EVs that contain signalling factors required for neuronal development are carried along in the embryonic and adult CSF, thereby reaching stem cell niches [24]. EV-borne components are insulin-like growth factor [44], folate receptor-α and pro-inflammatory miRNA such as miRNA-146a and miRNA-155. These CP-derived EVs are not only transported by CSF but can also cross the ependymal cell layer and reach brain parenchyma cells [45,46]. One significant advantage of EV-borne signalling factors is that they can be specifically labelled with fluorescent antibodies directed against EV transmembrane proteins and be traced by live fluorescence microscopy in space and time. Despite much research activity, EV research results are at times ambiguous owing to differences in purification methods, EV heterogeneity, heterogeneous sources and influence of growth conditions on the cargo of EVs [35,47–49]. Accordingly, it is of great importance to use reproducible and thorough methods for isolation, purification and characterization of EVs.

In summary, the CP is a rich source of signalling compounds, some of which are dissolved single molecules, while others are packaged into EVs or other types of lipid particles [49]. Because EVs can be labelled with fluorescent antibodies against the extracellular domains of EV transmembrane proteins, and because EVs can be visualized by fluorescence microscopy and nanoscopy, they are a CSF component amenable to the study of cilia-mediated transport in the ventricular system.

## 4. Cellular constituents of ependyma

Knowing the biology and biochemistry of the ependymal cells is crucial since these cells form the boundary between CSF and brain parenchyma [5,6]. Through their apical cilia bundles, they are capable of moving CSF, and they represent the first point of contact between CSF constituents and the brain. The ependyma of the v3v is predominantly made of E1 cells (ependymal cells) that are apically covered with microvilli, but also includes E2 and E3 cells [50]. E2 cells carry apically two (potentially motile) 9 + 2 cilia and are likely to be identical with α-tanycytes [50–53]. E3 cells are uni- or bi-ciliated and are better known as β-tanycytes. Cilia of E3 cells are thought to be sensory cilia. E3 cells are most abundant in an approximately rectangular patch of ependyma in the posterior most part of the v3v (black area in figure 2). E2 cells are also found in this patch and additionally are scattered throughout the posterior-most third of the v3v and in a stripe extending along the ventral aspect of the outflow tract [50]. Like E1 cells, E2 and E3 cells have their apical side directly exposed to CSF. Basally, tanycytes extend one or two long processes that reach deep into the brain parenchyma (figure 1; [51,53]) where they contact several hypothalamic nuclei as well as blood vessels.

The current literature discusses four types of tanycytes [42,52], but the tanycyte population may be even more diverse. α1-Tanycytes populate the upper half of the tanycyte patch and their processes project into the dorsomedial and ventromedial hypothalamic nuclei. α2-Tanycytes are located ventral to their α1-siblings and send processes to ventromedial and arcuate hypothalamic nuclei. When exposed to growth factors by intraventricular infusion, α1- and α2-tanycytes will proliferate and, depending on the nature of the infused factor, give rise to neurons, α1- and α2-tanycytes, astrocytes and oligodendrocytes [54,55]. Thus, factors delivered to the v3v through CSF are capable of triggering cell proliferation. Ventral to the α2-tanycyte region are β1-tanycytes that send processes to the arcuate nucleus and the median eminence. The CSF-contacting face of the median eminence is made of the apical face of type β2-tanycytes that carry sensory cilia. In juvenile mice, β-tanycytes proliferate and yield astrocytes and neurons targeted to the median eminence. β2-Tanycytes also proliferate to sustain a size expansion of the median eminence typically seen in juvenile animals [42,56]. β2-Tanycytes extend their basal protrusions towards the plexus of fenestrated blood capillaries that are part of the hypophyseal portal microcirculation. This contact of tanycyte protrusions with the capillaries could allow the transfer of blood-borne substances into the tanycyte and then into the CSF and *vice versa*. Tanycytes of the E3 type also populate the *organum vasculosum* of the *laminae terminalis* (OVLT) located at the anterior wall of the v3v. These tanycytes are in direct contact with v3v through the apical area of the tanycytes that sprouts sensory cilia [50,57].

Taken together, the wall of the third ventricle is histologically complex and is made of several cell types. The dominant cells are the ependymocytes that sustain a lifelong complex CSF flow. Tanycytes provide a conduit between ventricular CSF and the brain parenchyma. Tanycytes also form bona fide stem cell niches in the adult brain, and these niches respond to CSF-infused growth factors by proliferation and differentiation.

## 5. v3v exhibits complex cilia-driven flows

**Figure 3.**
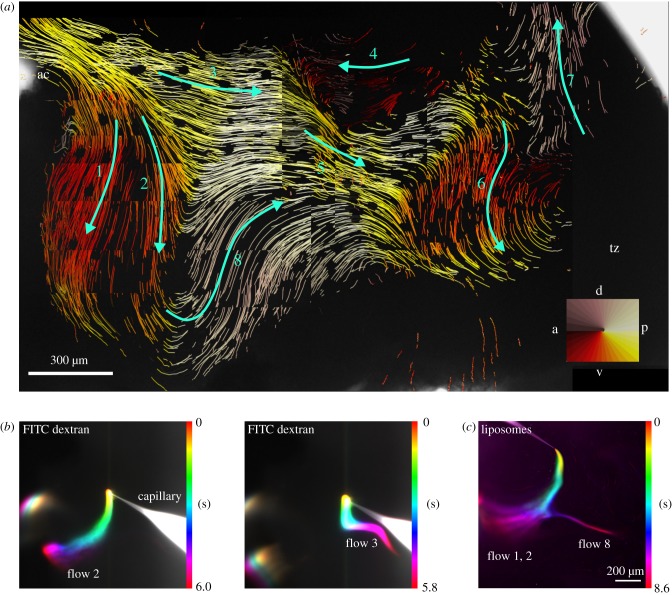
Flows in the v3v. (*a*) Flow map generated by particle tracking shows eight streams represented by green arrows. See colour compass for the flow direction colour code. Inflow and outflow ducts are on the top left and top right, respectively. The anterior commissure (ac) is a nerve fibre located below the inflow duct. Tanycytes (tz) do not carry cilia bundles and thus do not generate flow. Dorsal (d) is on the top and anterior (a) to the left. (*b*) Propagation of FITC-dextran and (*c*) fluorescently labelled liposomes in cilia generated near-wall flow. A capillary was used to inject small amounts of either FITC–dextran (*b*) or fluorescently labelled liposomes (*c*) into the inflow region of the v3v. The propagation was recorded over time and a temporal colour code applied using Fiji. The temporal colour code shows the change of the fluorescence intensity over time. The applied droplets of FITC-dextran and liposomes followed the cilia-generated streamlines. Raw data for (*b*) are from [2]. Dorsal is on the top and anterior to the left.

Does CSF flow through the ventricles like water would flow in a system of interconnected rivers and lakes? This is definitely not the case in the v3v. Explants of the ventricular walls show complex flow patterns that consist of eight distinct streams that are interconnected like roads in a map (figure 3*a*). To generate this flow map, 1 µm diameter fluorescent beads are added in bulk to the medium in which the v3v explants are kept. The movement of beads evoked by beating cilia bundles is captured and a particle tracking software is used to compute individual bead tracks [2]. These tracks are plotted on top of the v3v explant, resulting in a map of continuous, interwoven flows that represent fluid movement across the entire v3v surface. Alternatively, fluid flow can readily be visualized by locally applying fluorescein isothiocyanate (FITC)-dextran (figure 3*b*) or fluorescent liposomes (figure 3*c*) to the ependyma of the explant. Rates of transport of beads and liposomes across the ependyma are in the range of several hundred micrometres per second. Since in the mouse, the v3v is about 2 mm long, moving of CSF from the inflow to the outflow occurs within a few seconds. Either of these methods reveals a complex flow pattern in the v3v that consists of straight flows, bent flows and whirls capable of directional transport and of subdividing the ventricular volume [2]. These flows are entirely regulated by properly oriented cilia bundles. Cilia bundle orientation is genetically determined by planar cell polarity proteins [4,58–60].

## 6. Fluid dynamics

CSF flow in the ventricular system of mammalian brains is driven by CSF secretion into the ventricles and by motile cilia beating, and plays an important role in the delivery of CSF components and washout of waste [2]. CSF is not stagnant but pulsating *in vivo* owing to heart beating [15,17,61], respiration [14] and even head movement [62]. Different approaches have been used to investigate the CSF flow experimentally or numerically (see [63] and the references therein). Non-invasive MRI offers a way to evaluate the flow *in vivo* [14,61,62] but cannot provide detailed flow information owing to its limited spatial resolution, especially for the mouse brain. Particle tracking with microscopy is capable of recording cilia-induced flow networks along the wall in opened ventricles [2] but does not give the volumetric flow.

Alternatively, detailed CSF flow in the 3D ventricles can be investigated with computational fluid dynamics. Previous numerical studies focused on the human brain and investigated the CSF flow in idealized geometry or subject-specific geometry of ventricles reconstructed from medical imaging [64–67]. In most investigations, the effect of cilia on the flow was not studied. Recently, Siyahhan *et al*. [68] took into account the effect of the cilia beating pattern in the lateral ventricles of the human brain by applying a force density on the flow and studied the near-wall CSF flow dynamics. However, the cilia or beating directions were assumed to be aligned with the CSF net flow, which may not be true when there is a complex cilia pattern in the ventricles as we detect in our experimental investigation in the mouse (figure 3*a*) [2]. Interestingly, the Siyahhan *et al*. simulations [68] show that flow in the appendix of the human 3V had a very small oscillatory component, suggesting a prominent role of cilia there.

Compared with the human brain ventricles, the two parts (dorsal and ventral) of the 3V of the mouse brain are much more separated. The ventral part is also much narrower than its dorsal counterpart. As a result, most oscillatory CSF net flow will take the dorsal route owing to its smaller resistance and, therefore, the flow in the ventral part will be dominated by cilia beating.

## 7. Comparing flow directions in explants with the intrinsic translational polarity of the v3v

**Figure 4.**
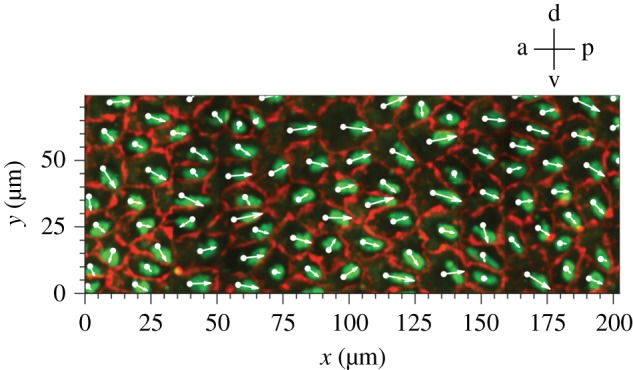
Translational polarity of ependymal cells in flow module 3. White arrows show the direction of translational polarization, which is determined by drawing a vector from the cell centroid to the centroid of the cilia bundle. Cell boundaries were detected with an anti-β-catenin antibody and basal bodies were stained with anti-γ-tubulin antibody.

Media-immersed v3v explants exhibit a complex flow pattern. What is the evidence that this pattern reflects the CSF flow *in vivo*? There are two lines of evidence that suggest that flow directions in explants and in the live animal are very similar. First, it was observed that in the lateral ventricle, cilia effective stroke and flow directions, as detected by particle tracking (figure 3*a*), are correlated [4]. In the v3v, flows 3 and 4 are directly opposing each other (figure 3*a*). The corresponding movies of cilia beating in the ependyma underneath flows 3 and 4 show that cilia beat in the opposite direction as well (Movie S4 in [2]). One could still argue that in explants cilia bundles have reoriented relative to their position in the brain. This implies that translational polarity would change upon dissection and/or transfer of the v3v tissue into the culture medium. Immunohistochemical staining of ependymal cells and of cilia bundles in native v3v ependyma allows one to determine translational polarity. Figure 4 shows translational polarity for E1 cells driving flow 3. The arrows represent translational polarity cell-by-cell point in their majority towards the posterior end of the v3v. Figure 3*a* shows that flow 3 is also pointed towards the posterior end of the v3v. The example discussed here suggests that there is a good match between flow direction in the explant and translational polarity in the corresponding region in the intact brain.

## 8. Outlook

Why is there a ventricle in the hypothalamus and what would be the purpose of a complex directional flow pattern in the v3v? The answer could be that CSF delivers factors to specific hypothalamic nuclei, especially those that are located at or near the basal side of ependyma. Ependymal flow 8 passes above the suprachiasmatic nucleus, which regulates the circadian clock. Flow 6 passes by the arcuate nucleus, which controls various metabolic functions. Tanycytes provide a direct exchange between CSF and neuronal or vascular targets. Flow 6 streams to the tanycyte region, while flow 7 leads away from the tanycytes. In the explant, there is no flow above the tanycytes, but in the closed ventricle in the brain, flows 6 and 7 would provide coordinate transport of CSF solutes to and then away from the tanycyte region.

This view of the ventricular system as a site of targeted flow provides a number of challenges. What are the substances that are being delivered? There are many factors in CSF, and they not only need to be identified but assays for a function in the hypothalamus need to be developed. These functions could relate to metabolism, circadian timing or the control of stem cell growth and differentiation. How can CSF constituents transit from CSF to their subependymal targets? For solutes such as growth factors, there may be specific receptors on E1, E2 or E3 cells. The uptake of EVs may involve endocytosis [69]. Where do signals come from? One likely source is the secretory epithelium of the CP, but it is unclear how signals coming from the lateral ventricles or the d3V are funnelled into the v3v. Could the divergent flows at the inflow act as a sorting device? Do the observed flows change rhythmically and do they change during the lifespan of an animal? That there is directional movement of solutes, signalling molecules and EVs in the fluid phases of the central nervous system (extracellular fluid and CSF) has long been known and has led to the concept of ‘volume transmission’ [70,71]. The case can be made that cilia-driven directional flows are a manifestation of volume transmission in that the ciliary conveyor belts provide the means of transportation.

Protein targeting mechanisms in cells may serve as a useful paradigm for thinking about the ventricular transport. A key insight for understanding protein targeting within cells was the realization that proper sorting and delivery relies on information encoded in the protein itself. In analogy, it is possible that EVs, potential carriers of CSF signals in the ventricular system, expose on their surface protein sequences that are used as a postal code for sorting into specific streams and for proper delivery to the apical face of specific E1, E2 and E3 cells. Addressing all these points experimentally is a daunting task. The reward of such an effort could be the discovery of a cilia motility-based signalling system in an important and ancient part of the brain.

## Data accessibility

This article has no additional data.
